# Examining Racial Discrimination Index and Black-Years of Potential Life Lost (YPLL) in South Carolina: A Real-Time Social Media Research

**DOI:** 10.1007/s40615-025-02416-7

**Published:** 2025-04-08

**Authors:** Yunqing Ma, Peiyin Hung, Xiaotong Shen, Zhenlong Li, Curisa Tucker, Jiajia Zhang

**Affiliations:** 1 Arnold School of Public Health, University of South Carolina, 915 Greene Street, Columbia, SC 29208 USA; 2 School of Statistics, University of Minnesota, Twin Cities, USA; 3 Department of Geography, The Pennsylvania State University, State College, PA 16801 USA; 4 College of Nursing, University of South Carolina, Columbia, SC 29208 USA

**Keywords:** Racial discrimination index, YPLL, South Carolina, Social media

## Abstract

**Purpose:**

Despite efforts to reduce health disparities, Black Americans still face higher mortality rates than Whites. Racism has been recognized as a significant social determinant of health. Using social media data, human-being qualitative coding, and AI, we created a county-level racial discrimination index, exploring its association with years of potential life lost (YPLL) rates.

**Methods:**

Through human-AI collaborative approaches using X/Twitter data, we calculated yearly county-level racial discrimination index (RDI)—number of racial discrimination posts per 100,000 in-county non-duplicated posts and examined the relationship between RDI terciles and YPLL per 100,000 non-Hispanic Black individuals. Annual data for the covariates were derived from 2018–2022 County Health Rankings and American Community Surveys across all South Carolina (SC) counties.

**Results:**

RDI increased from 2018 (mean [SD], 1.443 [1.991]) to 2022 (3.439 [5.761]). Adjusting for county sociodemographic factors and historical trends, RDI was associated with the YPLL rate (marginal effects, highest vs. lowest tercile, 421.3; 95% confidence interval, 134.7–709.8; *p* = 0.006).

**Conclusions:**

Digital racial discrimination was highly associated with Black YPLL rates, confirming the importance of racial discrimination in health disparity, especially premature deaths. Addressing explicit and implicit racism in highly affected counties is crucial for reducing persistent health inequities and promoting equity in communities.

## Introduction

In 2000, the Institute of Medicine (IOM) published the landmark report “To Err is Human,” one of the earliest acknowledgments, which brought national attention to systemic issues in the US healthcare system, including the role of racism in shaping racial health disparities [1, 2]. More than two decades later, Black Americans still experience disproportionately worse health outcomes, such as premature death [3, 4]. Over the past 20 years, Black Americans have experienced 1.63 million excess deaths and lost over 80 million years of life in contrast to their non-Hispanic White counterparts [3, 5]. Particularly, the average rate of years of potential life lost (YPLL) per 100,000 individuals over the past 22 years was 20,365 for White males and 15,428 for White females, while for Black males and females, it stood at 31,944 and 23,360, respectively [3, 6].

Persistent premature death disparities between Black and White Americans are multifaceted and often result from structural racism and discrimination [7]. Rooted in slavery, segregation, and ongoing racial biases, structural racism continues to shape health disparities facing Black populations [8–10], by limiting their access to resources and opportunities. Often, Black individuals were less likely than their White peers to have access to quality education for adequate health literacy, stable employment, safe housing, healthy food, and timely and culturally competent healthcare [11]. The scarcity of these resources and opportunities that promote health, combined with discrimination entrenched in some communities within American society, further perpetuated avoidable death and widen Black-White disparities in health outcomes and YPLL [12, 13].

Racism, in its essence, refers to the belief in the inherent superiority or inferiority of individuals based on their race, coupled with actions or policies that reinforce these beliefs [14]. It manifests in various forms, ranging from interpersonal racism, institutional racism, to structural racism [15–17]. Existing measures of racism focus on structural racism, such as Residential Segregation Index, and Structural Racism Effect Index. Specifically, Residential Segregation, which reflects the likelihood of Black residents interacting with White residents across Census tracts in a county, is commonly used to serve as a proxy for structural racism due to historical oppression [18]. The Residential Segregation Index quantifies this segregation by providing a measurable indicator of racial separation within communities. Structural Racism Effect Index (SREI) was established to measure the neighborhood-level impact of structural racism across the United States using publicly available data, including the American Community Survey (ACS), the U.S. Census Bureau Supplemental Poverty Measure, and the National Center for Health Statistics U.S. Small-area Life Expectancy Estimates Project (USALEEP) [19].

However, the conceptualization and operationalization of racism in health research often exhibit gaps, primarily due to the reliance on self-reported perceived discrimination or historical structural factors [20, 21]. Self-reported measures of perceived discrimination, while valuable in capturing individuals’ experiences, are inherently subjective and susceptible to various biases, including cognitive biases and social desirability [22]. Moreover, such measures typically only capture overt acts of discrimination that are consciously recognized by individuals, potentially overlooking more subtle forms of bias and systemic inequalities [22]. Additionally, self-reported measures of discrimination may lack the contextual information necessary to fully comprehend the underlying social and economic factors contributing to racial disparities in health [22, 23]. As a result, there is a need for more nuanced and comprehensive approaches to conceptualizing and measuring racism in health research, one that considers both individual experiences and broader structural determinants of health outcomes.

With social media being widely used, it has become a leading approach for communication and information exchange, despite ongoing debates regarding its authenticity and psychological impact [24, 25]. While social media platforms can sometimes present curated or distorted portrayals of life, they also serve as spaces where users share candid thoughts, engage in public discourse, and report personal experiences in real time, making them valuable data sources for social research. The utilization of social media data coupled with artificial intelligence (AI) algorithms presents a promising avenue for studying patterns of racial discrimination in real time. For instance, previous studies have utilized natural language processing (NLP) techniques, including transformer-based models such as bidirectional encoder representations from transformers (BERT), to classify hate speech, detect racial bias, and conduct sentiment analysis on large-scale social media data [26], while another study employed sentiment analysis to study the impact of racial bias in online discourse [27]. With over 300 million monthly users, the social media platform, X/Twitter, offers a wealth of up-to-date data on a large scale, providing researchers with unprecedented access to diverse perspectives and experiences [28]. Users often express their thoughts and opinions freely on these platforms, offering unfiltered insights into their attitudes and beliefs regarding race and discrimination [29, 30]. Despite the growing use of social media and AI to study racial discrimination, limited research has focused on the specific sociopolitical and historical context of South Carolina (SC). Furthermore, geotagged social media posts enable researchers to conduct geospatial analysis to uncover patterns of racism at various geographic levels. By leveraging advanced AI algorithms, researchers can analyze massive datasets efficiently, identifying trends, patterns, and outliers indicative of racial discrimination [31, 32]. Ultimately, the integration of social media data and AI technology has the potential to complement traditional methods of studying racial discrimination, offering a more comprehensive understanding of its prevalence, dynamics, and impact in contemporary society.

In this study, we leveraged social media data in conjunction with an AI algorithm to construct a comprehensive assessment of county-level racial discrimination and its association with YPLL rates among Black populations. We hypothesize that racial discrimination is one of the key factors in health inequities that contributes to premature deaths in Non-Hispanic Black populations.

## Methods

### Study Design and Data Sources

This cross-sectional ecological study employed three databases: first, using X/Twitter Application Program Interface, we extracted 11,562,191 tweets posted by individuals geotagged in South Carolina from 2018 to 2022. We excluded those from institutional accounts before identifying racial discrimination posts using human-being qualitative coding by two professionals in Excel, with inter-rater reliability assessed at each iteration of the coding process to ensure consistency, and using modern machine learning methods on natural language processing (NLP). Second, we derived county-level YPLL rate for all 46 counties in South Carolina [33] from 2018 to 2022 from County Health Rankings data. Third, American Community Surveys (ACS) data provided previously documented social determinants of health confounding factors for YPLL and racism: the percentage of individuals aged over 65 years, female individuals, individuals identifying as White or Black, residents in rural areas, age-adjusted mortality and life expectancy for the Black population, Black household income, the percentage of Black children living in poverty, homicide rate, and Residential Segregation Index. These variables were accounted for in the data analysis using multivariable linear mixed-effects models to assess their influence on the association between racial discrimination and YPLL.

### Measures

#### Racial Discrimination Posts

Our key exposure variable is the racial discrimination ratio, calculated as the number of racial discrimination-related posts per 100,000 total posts for each county in each year. Due to the substantially skewed distribution of the racial discrimination ratio, where a small number of counties exhibited disproportionately high values while many counties had near-zero values, we further logarithm transformed this racial discrimination ratio—hereafter, racial discrimination index. To analyze X/Twitter posts and determine their label of racial discrimination, we employed four phases (Appendix Fig. 1): (1) working with a domain-expert librarian, we conducted scoping literature review of several databases and engines from years 2003 to 2023, including CINAHL, PsychInfo, Medline, PubMed, urban dictionary websites, and general Internet searches. Ancestry searching and journal hand searching were also conducted, leading to the identification of 32 additional racism/discrimination measures. In total, 46 measures were considered, of which 27 met the predefined inclusion/exclusion criteria for this review and were included in the analysis. To identify racial discrimination-related posts, we screened for the presence of any of the 27 selected words or phrases that convey derogatory, racist, or insulting meanings toward Black or African American populations (Appendix Table 3). These words/phrases were identified based on linguistic research and expert consultation and were incorporated into the inclusion criteria for detecting racial discrimination-related content. (2) Professional labeling of 2500 posts was conducted by two researchers: one author (PYH), an Asian individual experienced in studying racism and discrimination, and another author (CT), a native English speaker who identifies as Black and is familiar with Black vernacular language. Both researchers received training in qualitative coding procedures and racial discourse interpretation. During this professional coding phase, we specifically identified the posts where Black individuals used racialized terms in a reappropriated or self-expressive way and did not consider them as racial discrimination. Inter-rater reliability during initial manual labeling was 89% and reached 100% after consensus discussions. (3) Constructing a BERT (Bidirectional Encoder Representations from Transformers) model. BERT [34], a state-of-the-art NLP model, has demonstrated remarkable effectiveness in various NLP tasks, including text classification [35]. Our BERT model was trained using a labeled dataset where each X/Twitter post was annotated with a binary label indicating whether it was related to racial discrimination or not from professionals. The training process involved fine-tuning the pre-trained BERT model. We utilized standard practices for model evaluation and hyperparameter tuning to ensure optimal performance [34]. Once trained, the BERT model was applied to classify new X/Twitter posts, to identify the instances of racial discrimination. (4) Professional validating a randomly selected 500 BERT labelled posts. The prediction accuracy of the NLP on identifying racial discrimination posts was over 95. Using the final trained model, we identified 362,627 racism posts by individuals residing across 46 counties in SC from 2018 to 2022.

#### YPLL Rates

Our key outcome variable is the county-level annual age-adjusted YPLL rate per 100,000 for non-Hispanic Black residents in a county per year.

### Statistical Model

The primary exposure variable in this study is the county-level racial discrimination index. We categorized each county into one of the three terciles based on their racial discrimination index per year. To facilitate comparison, we categorized all 46 counties in South Carolina into three terciles each year based on the distribution of the racial discrimination index: the lowest tercile (bottom 1/3, counties with the lowest racial discrimination index), the middle tercile (middle 1/3), and the highest tercile (top 1/3). Subsequently, we compared their county socio-demographic mix and the existing social determinants of health indicators from ACS data across these terciles. These indicators included the percentage of individuals aged over 65 years, female individuals, individuals identifying as White or Black, and residents in rural areas, as well as metrics such as age-adjusted Mortality and Life Expectancy specifically for the Black population. Additionally, we examined county-level Black household income, the percentage of Black children living in poverty, homicide rate, and segregation index. Two-group *t*-test was used for tercile mean comparison after normality test, and chi-squared test was used for categorical predictor comparison.

Unadjusted and multivariable linear mixed-effects models (LMMs) were used to examine the associations between various demographic and socioeconomic factors and the YPLL rate among Black populations. All models controlled for county-level random effects using the FIPS code and yearly random effects. Estimates and 95% confidence intervals (CI) for estimated coefficients were calculated. LMMs were fitted using the *lme4* package in R 4.1.2.

## Results

From Table 1, racial discrimination increased in general from 2017 to 2022, especially for the Tercile III (one-third of the counties with the largest ratio rates), the ranges of the rates raised from 0.494–5.580 (mean, 1.630, standard deviation (standard deviation, SD) 1.400) to 1.403–20.925 (mean, 9.328; SD, 7.016). Percentage of individuals aged over 65 years, female individuals, and individuals identifying as White or Black showed no significant difference among different terciles. However, a greater percentage of residents in rural areas (69.5% vs 25.1%), age-adjusted mortality for Black people (620.1 vs 544.3 per 100,000 population), percentage of Black children living in poverty (43.0% vs 33.5%), and homicide rate (14.5% vs 8.3%), household income for Black people ($30,472 vs $40,733), and segregation index (32.6 vs 44.3) were related to greater racial discrimination.

**Table 1:** Demographics and county health outcomes in 2017 and 2022 by racial discrimination index tercile

	Tercile tweeter racial discrimination index				Tercile tweeter racial discrimination index			
	01/01/2017–12/31/2017				01/01/2022–12/31/2022			
	Tercile I	Tercile II	Tercile III	*P*-values	Tercile I	Tercile II	Tercile III	*P*-values
**Number of counties**	10	11	11		9	9	9	
**Positive ratio, min–max**	0.071**–**0.134	0.134**–**0.470	0.494**–**5.580		0.217**–**0.398	0.444**–**0.981	1.403**–**20.925	
**Positive ratio, mean (SD)**	0.111 (0.019)	0.250 (0.110)	1.630 (1.400)		0.342 (0.058)	0.647 (0.181)	9.328 (7.016)	
County mean age								
% 65 +	16.821 (4.410)	18.046 (2.823)	18.602 (0.452)	0.364	19.645 (4.576)	18.446 (3.753)	19.877 (1.464)	0.807
**Gender**								
%Female	51.153 (0.594)	51.774 (0.890)	51.068 (2.238)	0.868	51.809 (0.620)	51.449 (0.814)	50.767 (1.979)	0.092
**Race/ethnicity**								
%White	72.546 (11.455)	67.756 (15.176)	52.052 (24.757)	0.091	68.514 (8.206)	63.973 (15.863)	49.664 (23.281)	0.106
% Black	21.016 (10.820)	27.523 (15.358)	43.111 (26.548)	0.058	20.097 (9.297)	24.430 (16.464)	40.967 (26.993)	0.109
**County location**								
% Rural	22.299 (9.028)	44.233 (14.542)	70.211 (7.152)	** < 0.001**	25.068 (9.814)	35.406 (16.292)	69.515 (8.205)	** < 0.001**
**Majority rural community**								
Yes	0	3	11		0	1	9	
No	10	8	0		9	8	0	
**County health outcomes**								
Age-adjusted mortality (Black)	497.388 (57.238)	524.441 (64.561)	601.173 (65.519)	** < 0.001**	544.341 (78.998)	515.118 (69.284)	620.815 (87.237)	**0.065**
Life expectancy (Black)	75.176 (1.450)	74.425 (1.281)	72.836 (1.652)	** < 0.001**	73.936 (1.804)	74.583 (1.636)	72.513 (2.081)	0.130
Black household income	34,921.400 (4594.388)	31,631.364 (4846.529)	26,921.545 (4127.266)	** < 0.001**	40,733 (5378.005)	41,743.778 (7016.439)	30,472.222 (6146.068)	**0.004**
% Children in poverty (Black)	34.945 (9.591)	41.774 (9.056)	44.631 (10.138)	**0.027**	33.532 (4.433)	33.362 (10.476)	42.957 (11.255)	**0.043**
Homicide rate	6.810 (1.980)	7.282 (2.029)	15.000 (6.162)	** < 0.001**	8.297 (2.739)	8.932 (2.093)	14.468 (8.793)	**0.025**
Segregation index	41.878 (6.857)	33.297 (8.340)	25.520 (6.649)	** < 0.001**	44.322 (5.702)	41.089 (10.820)	32.625 (11.277)	**0.015**

Adjusting for socio-demographic and county random effects (Table 2), racial discrimination index (346.56; 95% CI, [19.25, 654.80]), the percentage of population that is female (46.84; 95% CI, [21.14, 71.88]), and the percentage of population living in a rural area (3.10; 95% CI, [0.71, 5.42]) were associated with increased YPLL rates. From the LMM adjusting for demographics and socio-economic predictors, logarithm of positive ratio index (461.34; 95% CI, [132.06, 773.24]), the percentage of population that is female (47.51; 95% CI, [19.64, 74.71]), and that is unemployed (18.36; 95% CI, [6.12, 30.80]) were associated with an increasing YPLL.

**Table 2:** Odds ratios from unadjusted and multivariable linear mixed-effects models

Characteristics	Unadjusted model	Multivariable model
Logarithm of racial discrimination index	679.54 (417.36, 942.49)*	461.34 (132.06, 773.24)*
Percentage of population that is female (per 10 percentage)	29.75 (− 1.65, 61.78)	47.51 (19.64, 74.71)*
Percentage of population living in a rural area (per 10 percentage)	4.39 (2.54, 6.25)*	2.79 (− 1.03, 6.17)
Percentage of population that is Black (per 10 percentage)	5.86 (3.36, 8.33)*	− 6.72 (− 24.84, 12.67)
Percentage of population that is Non-Hispanic White (per 10 percentage)	− **6.13 (**− **8.97,** − 3.30)*	− 8.95 (− 27.59, 10.94)
Percentage of adults under age 65 without health insurance (per 10 percentage)	5.07 (− 14.06, 24.28)	− 3.58 (− 21.75, 17.90)
Percentage of unemployed adults age 21 and over (per 10 percentage)	19.80 (6.69, 33.61)*	18.36 (6.12, 30.80)*
Percentage of adults age 25 and over with a high school diploma or equivalent (per 10 percentage)	7.12 (2.88, 11.09)*	2.95 (− 3.99, 10.18)
Household income for the Black people (unit: $1000)	33.31 (− 53.39, 105.30)	60.52 (− 13.73, 121.11)

Notes: models control for county-year random effects. Coefficients for all covariates’ crude model were calculated *without* adjusting other covariates

## Discussions

To our knowledge, this is the first study to leverage social media data to identify racial discrimination-related terms, slurs, and phrases using a novel natural language processing (NLP)-generated racial discrimination index. Comparing the final validated racial discrimination index with traditionally well-adopted structural racism indicators, we found significant associations between this human-AI-collaborated racial discrimination index measure with higher percentages of Black children living in poverty (43.0% vs 33.5%), higher Black-White segregation index (32.6 vs 44.3), and lower Black Household income ($30,472 vs $40,733). Crucially, after controlling for these structural factors, the racial discrimination index remained associated with increased YPLL (OR, 461.34; 95% CI, [132.06, 773.24]), reinforcing the harmful impact of racial discrimination on health outcomes.

The findings of this study underscore the pervasive influence of racial discrimination on health outcomes among Black populations in SC. These results align with existing literature emphasizing the role of structural racism [36] and socioeconomic disparities in perpetuating health inequities among racially marginalized communities [37, 38]. Importantly, our study extends beyond traditional measures of racism by utilizing social media data and AI to capture real-time expressions of racial discrimination. The use of AI models provides a unique advantage, offering a cost-effective, easily accessible, and continuously updated tool for capturing the dynamic and evolving nature of discriminatory language, which traditional structural data may not readily reflect. By acquiring the huge amount of information available on social media platforms like X/Twitter, we gained insight into the prevalence and dynamics of racial discrimination across individuals posting in each county. This approach offers a novel and comprehensive method for studying racism in contemporary society, complementing traditional research methods and providing a clearer understanding of its impact on health outcomes.

The concept of YPLL serves as a powerful tool for quantifying the extent of health inequities experienced across the entire lifespan [3]. YPLL offers insights into the burden of chronic diseases and conditions [39], and sheds light on disparities in maternal and child health [40], instances of violence [41], and various other preventable causes of death. Moreover, YPLL can also be extended to reflect various socioeconomic factors, including disparities in access to education, employment opportunities, housing stability, and quality healthcare services [42]. Thus, YPLL serves as an important reflection of racial discrimination and disparity within healthcare and broader societal systems [43].

The implications of our findings are manifold. Firstly, targeted interventions aimed at addressing interpersonal racism and socio-economic disparities to improve population health outcomes among Black communities are urgently needed [44, 45]. This necessitates not only dismantling institutional barriers and ensuring equitable access to healthcare and resources but also confronting the pervasive issue of digital bullying and discrimination. By recognizing the detrimental impact of online harassment and bias on mental well-being, access to essential resources, healthcare disparities, and the intersectional nature of discrimination, interventions must extend beyond traditional realms of systemic oppression to encompass digital spaces [46, 47]. Additionally, our study highlights the potential of social media data and AI technology as valuable tools for monitoring and addressing racial discrimination in real time [48, 49]. By leveraging these innovative approaches, policymakers, public health officials, and researchers can develop more effective strategies for promoting equity and social justice.

However, it is essential to acknowledge the limitations of our study. First, the measured words/phrases listed in Appendix Table 3 that were labeled as racist are not exhaustive, and it is possible that relevant terms may have been omitted. Second, while manual labeling involved only two trained researchers, their domain expertise and the high level of inter-rater reliability reduced the likelihood of subjective bias. While social media data offer valuable insights into racial discrimination, they also present challenges, as some underserved individuals might not have access to these social media, e.g., the geotagged X/Twitter data might not be fully representative of the entire population groups [50]. Among those who did, we still found that rural communities had a higher racial discrimination index. In addition to the issues of representativeness, discriminatory remarks expressed using non-discriminatory terms and the uneven posting behavior of people with discriminatory views on social media further complicate the interpretation and generalizability of our findings. Furthermore, our analysis focused on South Carolina, limiting the generalizability of our findings to other geographic regions. Yet, just in one state, we have analyzed 11,562,191 posts with multiple iterations of validation processes. Future research should explore the applicability of our methodology in diverse contexts such as other racial/ethnic groups and other health outcomes and examine the effectiveness of interventions aimed at eliminating racial discrimination and improving health outcomes among marginalized populations.

## Conclusion

This social media research study contributes to the growing literature on racism and health disparities by offering a novel approach to studying racial discrimination via social media data and its relationship with population health outcomes. By integrating social media data and AI technology into public health research, we can understand the communities where digital racial discrimination has been occurring and intervene in a timely manner. The results that the developed racial discrimination index was strikingly associated with life expectancy and other socio-economic indicators demonstrate the promises of leveraging this algorithm to identify minority populations in need of support and provide the opportunity to reduce future discrimination-associated life loss.

## Figures and Tables

**Fig. 1:**
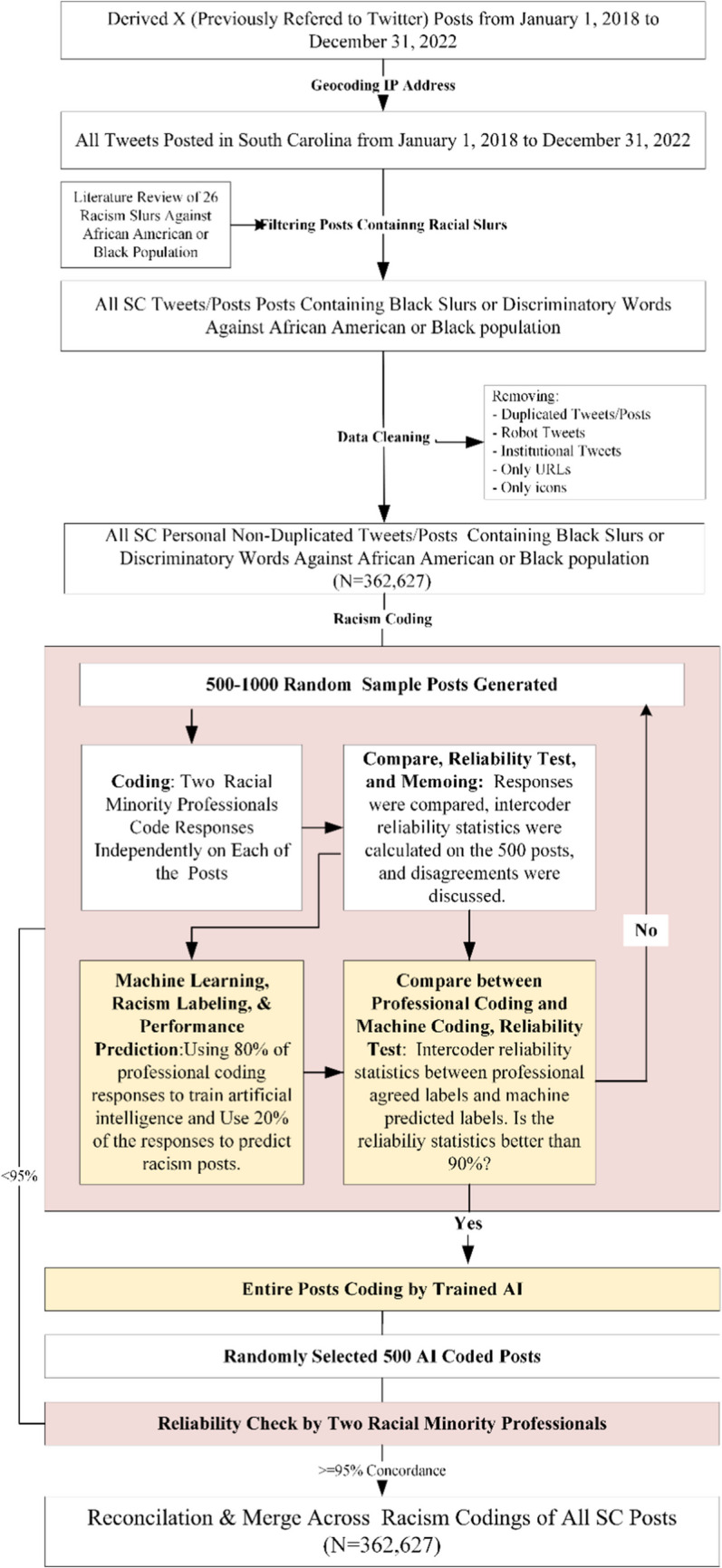
Flow chart for the process of defining discriminatory posts

## Data Availability

The final aggregated social media dataset used in the current study is available from the corresponding author upon request.

## Appendix

Figure 1

Table 3

**Table 3:** Words/phrases used in the United States that might have implications to look down, derogatory, racist, or insult each of the following groups on the basis of race, ethnicity

Race	Terms	Meaning, origin, notes	Reference
**Black**	Ape	Referring to outdated theories ascribing cultural differences between racial groups as being linked to their evolutionary distance from chimpanzees, with which humans share common ancestry Referring to Black individuals as Ape is an offensive and derogatory act that insults both their intelligence and appearance	Bradley, James (30 May 2013). “The ape insult: a short history of a racist idea.” The Conversation. Retrieved 11 April 2015 Spears, Richard A. (2001). *Slang and Euphemism: A Dictionary of Oaths, Curses, Insults, Ethnic Slurs, Sexual Slang and Metaphor, Drug Talk, College Lingo, and Related Matters*. Signet. ISBN 978–0 - 451–20,371- 7. p. 10
	Afro engineering, African engineering or n***** rigging	Shoddy, second-rate or unconventional, makeshift workmanship. Indirectly refers to black American people as worse or lower-valued than white American people when associating anything bad with them	Green, Jonathon (2005). *Cassell*’*s Dictionary of Slang*. Weidenfeld and Nicolson. ISBN 978–0 - 304–36636 - 1. P. 10 Poteet, Jim; Poteet, Lewis (1992). Car and Motorcycle Slang. p. 14, Afro engineering. ISBN 978–0 - 595–01080 - 6
	Alligator bait/gator bait	Target—Black people, especially black children First used in the early twentieth century, although some hypothesize the term originated in the late nineteenth century. The term derives from the fact that, during the slave trade, black children and babies were supposedly used as bait by whites in the USA to catch alligators	Spears, Richard A. (2001). *Slang and Euphemism: A Dictionary of Oaths, Curses, Insults, Ethnic Slurs, Sexual Slang and Metaphor, Drug Talk, College Lingo, and Related Matters*. Signet. ISBN 978–0 - 451–20,371 - 7. P. 6 Daniels, Peter (2017). Honest Conservatism Redirecting 50 Years of Black Voting. Page Publishing. p. 20. ISBN 978–1 - 63,568–158 - 1
	Aunt Jemima/aunt Jane/aunt Mary/aunt Sally	Target—Black women A black woman who “kisses up” to whites, a “sellout,” female counterpart of Uncle Tom	Green, Jonathon (2005). *Cassell*’*s Dictionary of Slang*. Weidenfeld and Nicolson. ISBN 978–0 - 304–36636 - 1. P.41–42
	Black buck/black brute	Target—Black man Originating in the post-Reconstruction USA , it was used to describe Black men who absolutely refused to bend to the law of white authority and were seen as irredeemably violent, rude, and lecherous	Laufs, Stefanie (October 2013). Fighting a Movie with Lightning: “The Birth of a Nation” and the Black Community. Diplomica Verlag. p. 56. ISBN 978–3 - 95,489–151 - 1
	Brillo Pad	Used to refer to the hair texture of a Black person	Natalie Morris (23 March 2022). “Black woman calls out white man who approached her in the gym and compared her hair to a mop.” Metro
	Buckwheat	The name of a black character that appeared in the Our Gang (Little Rascals) short films. Today it is used to refer to the hair texture of a Black person	ANDERSON, JAMES (6 May 2021). “Colorado GOP lawmaker who used racist term is reprimanded.” Associated Press Dareh Gregorian (7 May 2021). “Colorado GOP lawmaker reprimanded after calling colleague ‘Buckwheat.’” NBC News
	Burrhead/burr-head/burr head	Referencing Afro-textured hair	Green, Jonathon (2005). *Cassell’s Dictionary of Slang*. Weidenfeld and Nicolson. ISBN 978–0 - 304–36636 - 1. P 216
	Choc-ice	A person who is figuratively “black on the outside, white on the inside.”	Dilichi Onuzo (17 July 2012). “Is choc ice the new N-word?” “Rio Ferdinand fined for Ashley Cole ‘choc ice’ tweet.” *BBC Sport*. 17 August 2012
	Coon	Slur popularized by Coon songs played at Minstrel show. Originally associated in the 1830 s with the U.S. Whig Party who used a raccoon as their emblem. The Whigs were more tolerant towards blacks than other main parties. After the party folded the term “coon” evolved from political slang into a racial slur. Within African American communities, the word has been used to refer to a black person who is allegedly a “sellout”	“Van Jones on being called a ‘sellout’: ‘I’m more worried about outcomes than outrage.’” *TheGrio*. 27 November 2019. Retrieved 10 March 2022 “Staging Race—Karen Sotiropoulos.” *Hup.harvard.edu*. Retrieved 9 March 2022 Harper, Douglas. “coon.” Online Etymology Dictionary. Retrieved 1 November 2013 “Slavery in America.” Archived from the original on 13 February 2008. Retrieved 1 November 2013
	Golliwog	An expression which originally was children’s literature character and type of black doll but which eventually came to be used as a jibe against people with dark skin	“Thatcher axed by BBC’s One Show.” *BBC News*. 4 February 2009. Retrieved 1 November 2013
	Groid	Derived from “negroid.”	“An Accused Cop Killer’s Politics.” *Slate*. 10 April 2009. Retrieved 1 November 2013
	Jigaboo, jiggabo, jigarooni, jijjiboo, zigabo, jig, jigg, jigger	Target—Black people with stereotypical black features (e.g., dark skin, wide nose, and big lips) From a Bantu verb *tshikabo*, meaning “they bow the head docilely,” indicating meek or servile individuals	“jigaboo*.*” Oxford English Dictionary (*Online ed*.)*. *Oxford University Press. Retrieved *3 June* 2018*.* (Subscription or participating institution membership required.) Ayto, John; Simpson, John (2010). *Oxford Dictionary of Modern Slang*. Oxford University Press. ISBN 978–0 - 19–923,205 - 5 Holloway, Joseph E, ed. (13 July 2005). Africanisms in American Culture: jiggabo. ISBN 978–0 - 253–21,749 - 3. Retrieved 1 November 2013
	Jim Crow	SLANG n.: during the Reconstruction period of the USA , which followed the Civil War, an equally offensive and racist term as modern day “n*****”	Partridge, Eric (2006b). *The New Partridge Dictionary of Slang and Unconventional English: J-Z*. Taylor and Francis. ISBN 978–0 - 415–25,938 - 5. P.518
	Jungle bunny	Another word to define an African American. Slang; or, a black person in a white community	Ayto, John; Simpson, John (2010). *Oxford Dictionary of Modern Slang*. Oxford University Press. ISBN 978–0 - 19–923,205 - 5
	Mau-Mau	derived from Kenyans of the Kikuyu tribe involved in the Mau Mau Rebellion in the 1950 s	Fair Employment Practice Cases–Volume 20. Bureau of National Affairs. 1979. p. 723
	Mulignan/Mulignon/Moolinyan	Used by Italian-Americans. Deriving from “mulignana” the word for eggplant in some South Italian linguistic variants.[340] Also called a mouli	“Moolinyan.” *Lexico*. Oxford. Archived from the original on 1 October 2021 Richard Greene, Peter Vernezze, The Sopranos and Philosophy: I Kill Therefore I Am, Open Court Publishing, 2004, p. 162 Ayto, John; Simpson, John (2010). *Oxford Dictionary of Modern Slang*. Oxford University Press. ISBN 978–0 - 19–923,205 - 5
	N***** toe	A slur that is actually referring to a Brazil nut	“Explorations in Black Leadership–Reflections on Brown.” University of Virginia. Retrieved 28 June 2020
	Negroid/Negro	A derogatory term for an african american cyborg, commonly used in graphic novella such as 2000 A.D	urbandictionary.com
	N***	A word that has caused racial controversy over the last few decades and up to current times. It came from the derogatory term for African Americans “n*****” yet it has a completely different definition than said term. Often used as a slang term within the Black community	urbandictionary.com
	Oreo	Used as early as the 1960 s. Refers to a black person who is perceived as acting white, and therefore black on the outside and white on the inside like an Oreo cookie	Wilmore, Gayraud S. (1989). African American Religious Studies: An Interdisciplinary Anthology. Duke University Press. p. 441. ISBN 978–0 - 8223–0926 - 0. Retrieved 30 May 2014 Spitzberg, Irving J.; Thorndike, Virginia V. (1992). Creating Community on College Campuses: Beyond the Cultural Politics of Enjoyment. SUNY Press. p. 35. ISBN 978–0 - 7914–1005 - 9. Retrieved 30 May 2014 Boggs, Grace Lee (1998). Living for Change: An Autobiography. University of Minnesota Press. p. 117. ISBN 978–1 - 4529–0330 - 9. Retrieved 30 May 2014
	Uncle Tom	Refers to Black people perceived as behaving in a subservient manner to white authority figures	Herbst, Philip H. (1997). The Color of Words: An Encyclopaedic Dictionary of Ethnic Bias in the United States. Yarmouth Me: Intercultural Press. ISBN 978–1 - 877,864–97 - 1
	Sheboon	Target—black woman	Schmidt, Samantha (10 August 2019). “Federal judge awards over $700,000 to former American University student targeted in neo-Nazi ‘troll storm.’” Washington Post. Retrieved 27 February 2020
	Shine	Derived from shoeshiner, a lowly job many black people had to take	Green, Jonathon (2005). *Cassell’s Dictionary of Slang*. Weidenfeld and Nicolson. ISBN 978–0 - 304–36636 - 1. P.1265
	Smoked Irish/smoked Irishman	A nineteenth-century term intended to insult both blacks and Irish but used primarily for black people	Spears, Richard A. (2001). *Slang and Euphemism: A Dictionary of Oaths, Curses, Insults, Ethnic Slurs, Sexual Slang and Metaphor, Drug Talk, College Lingo, and Related Matters*. Signet. ISBN 978–0 - 451–20,371 - 7.p.118
	Sooty	Originated in the 1950 s	Ayto, John; Simpson, John (2010). *Oxford Dictionary of Modern Slang. Oxford University Press*. ISBN 978–0 - 19–923,205 - 5
	Toad	Prison slang	Partridge, Eric (2006b). *The New Partridge Dictionary of Slang and Unconventional English: J-Z*. Taylor and Francis. ISBN 978–0 - 415–25,938 - 5. P.786
